# Commentary: Racism and structural violence: Interconnected threats to health equity

**DOI:** 10.3389/fpubh.2022.958436

**Published:** 2022-09-06

**Authors:** Ekland Abdiwahab, Alice Guan, Cindy Hong, Scarlett Lin Gomez

**Affiliations:** ^1^Department of Epidemiology and Biostatistics, University of California, San Francisco, San Francisco, CA, United States; ^2^Independent Researcher, San Francisco, CA, United States

**Keywords:** racism, public health, health equity, public health critical race praxis, social justice policy

## Introduction

Sharif et al.'s recent publication in “Frontiers in Public Health” highlights the need for collective efforts to address structural racism and violence (1). We extend this work by emphasizing the need for solidarity between Black and Asian Americans in strategies to combat anti-Asian racism^1^. During the Civil Rights movements, immigration policies in the United States shifted to favor immigrants with “exceptional” abilities and high levels of education (2), leading to mass media narrative constructions of the “model minority” myth, largely spearheaded by white Americans. This myth simultaneously hyperbolized Asian American exceptionalism while advancing anti-Blackness (3, 4). Despite historic tensions between these communities, there is also a history of solidarity building. For instance, joint efforts between Black and Japanese Americans in the 1960s led to the repeal of the Emergency Detention Act, which allowed the federal government to preventively incarcerate individuals suspected of espionage (5). This example illustrates how historical solidarity efforts between these communities has led to changes in politics, which evidence suggests can impact tangible indicators of population health (6). We use Camara Jones' theoretic framework (7) to describe how internalized, personally mediated, and institutionalized racism and anti-Blackness can hinder contemporary solidarity efforts.

## Internalized racism, anti-Blackness, and colorism

Jones defines internalized racism as, “acceptance by members of the stigmatized races of negative messages about their own abilities and intrinsic worth” (7). Internalized racism, anti-Blackness, and colorism in Asian American communities create critical challenges to solidarity. Origins of colorism in Asian American communities are varied—while colorism among Asian Americans with European colonial histories likely valued lighter skin as a proximity to whiteness, colorism among Asian Americans without this history are instead rooted in perceptions of lighter skin as a proximity to wealth (8–12). In contemporary times, contributors of colorism are more complex, and cannot be disentangled from the globalization of western beauty ideals, which are created, commodified, and disseminated *via* global mass media often spearheaded by wealthier nations and center white beauty and white aesthetics as the global status quo. Across Asian countries, the billion-dollar skin lightening industry (13–15), serves as just one example of continued reinforcement of wealth, beauty, power, and sophistication associated with whiteness (8). Additionally, economic stability often requires alignment with power and whiteness as a tactic for survival and securing generational wellbeing, hindering solidarity between Asian and Black Americans. Racism and capitalism are interconnected systems (16), illustrated by the social and economic advantages based on proximity to whiteness, which has long term impacts on educational attainment, occupational status, income, mate selection, economic mobility, and ultimately health (8, 17–22). For Asian American new immigrants, interest group theory might help explain a desire for alignment with whiteness and rejection of Blackness (23).

## Personally mediated racism

Jones defines personally mediated racism as, “prejudice and discrimination, where prejudice means differential assumptions about the abilities, motives, and intentions of others according to their race, and discrimination means differential actions toward others according to their race.” Interpersonal anti-Blackness can be reflected within and between Asian groups (i.e., colorism) and perpetrated by Asian Americans against Black Americans, manifesting as negative assumptions about Black intelligence/abilities, and policing Black bodies in Asian American-owned businesses. Personally mediated anti-Blackness also hurts Asian Americans. Colorism often results in economic immobility for Asian Americans, i.e., those with darker complexion encounter more economic disadvantage (18, 19) and worse short-and long-term mental health outcomes (20).

## Institutionalized racism

Jones defines institutionalized racism as, “differential access to the goods, services, and opportunities of society by race.” One example of institutionalized anti-Black racism in Asian America is the systematic exclusion of Black ownership from the hair extension market. Currently, Korean Americans own the majority of Black beauty supply stores and control the manufacturing and distribution of hair extensions, restricting opportunities for Black Americans to participate in this market (24–27). Racial liberation requires solidarity across economic lines, and while Black-Korean communities have made critical steps to building faith and trust in each other (28), efforts to make systemic changes to the economic distribution of resources and opportunities including access to affordable housing, equitable access to social resources (e.g., affirmative action policies), anti-discrimination laws, and police reform, are critical to combat institutionalized racism against both groups. As another example, white dominance in media has played an important role in perpetuating institutional racism by obscuring intersectional Black-Asian solidarity efforts in favor of dividing narratives. Prevailing mass media narratives implicate Black men as primary perpetrators of anti-Asian violence, despite evidence which found the majority of perpetrators of anti-Asian incidents to be White men when race was reported (29). The media saturation of images of “Black on Asian” violence exists in the general media landscape which systematically over-reports Black crime while systematically under-reporting white crime (30), enforcing harmful stereotypes against Black people (31, 32) and fueling Black-Asian conflict. As a final example, institutionalized racism impacting both Black and Asian Americans can be observed in discourse about affirmative action policies, which scapegoat Black Americans as undeserving and Asian Americans as victims, even though a large proportion of Asian Americans support these policies.

## Discussion and contemporary examples

Despite these challenges, contemporary examples of solidarity exist. Asian Americans have made efforts to address anti-Blackness and understand its connection to anti-Asian racism (e.g., Letters for BLM, Asians for Black Lives) (33). Additionally, there are examples of organizations which center decarceral approaches in solutions to violence against Asian Americans (e.g., AAPI Civic Engagement Fund, Compassion in Oakland, Stop AAPI Hate). Ultimately, policies built on interconnectedness are critical to the mission of health equity. While acknowledging differences between Asian American ethnic groups, laws that were passed to address racism against Black Americans have also benefited Asian Americans. The 14th Amendment, the Civil Rights Act of 1964, the Voting Rights Act of 1965, and the Civil Rights Act of 1968 established citizenship and voting rights, desegregated schools, and outlawed race-based employment and housing discrimination (34–37). Resultant laws have increased Black and Asian American's social mobility (e.g., access to higher education/employment, voting, and homeownership). We implore public health scholars to acknowledge that racial and economic hierarchies rely on division between communities, and that long-term, sustainable solutions to anti-Asian racism must be aligned, and not against, the liberation of Black communities.

## Author contributions

EA and AG conceptualized the paper. EA, AG, and CH prepared the first draft of the manuscript. SG reviewed the abstract and manuscript. All authors approved the last draft of the manuscript.

## Funding

This work was supported by a grant from the National Cancer Institute (PI AG) (F31CA257351). This work was also supported by institutional funding for EA (UC San Francisco, Department of Epidemiology and Biostatistics).

## Conflict of interest

The authors declare that the research was conducted in the absence of any commercial or financial relationships that could be construed as a potential conflict of interest.

## Publisher's note

All claims expressed in this article are solely those of the authors and do not necessarily represent those of their affiliated organizations, or those of the publisher, the editors and the reviewers. Any product that may be evaluated in this article, or claim that may be made by its manufacturer, is not guaranteed or endorsed by the publisher.

## Footnotes

^1^For the purpose of writing this commentary, we will use the terms Black and Asian American to broadly describe the political and social experiences of these communities. However, we acknowledge that a tremendous diversity of ethnic groups, cultural practices, language, and traditions exist in both of these communities, and recognize that the social construction of race in the United States cannot be disentangled by the political and economic context against which these categories were established.
